# Role of mir-33a, mir-203b, mir361-3p, and mir-424 in hepatocellular carcinoma

**DOI:** 10.3906/sag-2004-214

**Published:** 2021-04-30

**Authors:** Burhanettin YALÇINKAYA, Esra GÜZEL TANOĞLU, Didem TAŞTEKİN, Sadrettin PENÇE

**Affiliations:** 1 Scientific and Technological Research Council of Turkey (TÜBİTAK) National Metrology Institute (UME), Kocaeli Turkey; 2 Aziz Sancar Institute of Experimental Medicine, İstanbul University, İstanbul Turkey; 3 Institute of Health Sciences, University of Health Sciences, İstanbul Turkey; 4 Institute of Oncology, İstanbul University, İstanbul Turkey; 5 Department of Physiology, Faculty of Medicine, İstanbul Medeniyet University, İstanbul Turkey

**Keywords:** Mir-33a, mir-203b, mir361-3p, mir-424, HCC, qRT-PCR

## Abstract

**Background/aim:**

Hepatocellular carcinoma (HCC) is one of the most aggressive cancer types. MicroRNAs (miRNAs) are small noncoding regulatory RNAs that function posttranscriptionally. miRNA deregulation was observed in the development and progression of HCC. In this study, we aimed to investigate the expression levels of four miRNAs (mir-33a, mir-203b, mir361-3p, and mir-424) in HCC patients in comparison to healthy individuals.

**Materials and methods:**

Venous blood samples were collected from both HCC patients and healthy individuals. In order to determine the relative expression levels of mir-33a, mir-203b, mir361-3p, and hsa-mir-424 in HCC patients, probe-based quantitative real time PCR (qRT-PCR) was performed. The cycle threshold (Ct) results were analyzed according to the 2−∆∆Ct method and statistical analyses were performed by SPSS Statistics version 15 for Windows.

**Results:**

qRT-PCR analysis revealed that the expression levels of mir-33a (fold change: 7.3 and P < 0.001), mir-203b (fold change: 4.6 and P < 0.001), and mir361-3p (fold change: 5.1 and P < 0.001)were downregulated compared to healthy individuals and mir-424 did not show any significant change between HCC patients and controls.

**Conclusion:**

Our results indicated that mir-33a, mir-203b, and mir-361-3p may significantly contribute to tumor pathogenesis in HCC and have potential to be used as a noninvasive biomarker for cancer therapy.

## 1. Introduction

HCCis the leading cause of cancer-related deaths worldwide. It is caused by hepatocytes and it is one of the most frequently diagnosed types of primary liver cancer, constituting 80%–90% of primary liver tumors [1,2]. Infection with chronic viral hepatitis B and hepatitis C is the leading etiological factor for developing HCC. It has many different risk factors including hereditary, exposure to chemical agents such as aflatoxin B1, smoking, and chronic alcohol abuse [3]. miRNAs are single-stranded, small noncoding post transcriptional regulator RNAs that are comprised of ~20 nucleotides and have an important role cancer progression [4].

The analysis of miRNA expression levels in blood provides critical information about the prognosis of the diseasessuch as cancer and monitoring of treatment. The analysis of tissue-specific miRNA expression level can both detect tumor origin and allow early diagnosis of carcinogenesis. Numerous studies have shown that circulating miRNAs have clinical importance as diagnostic and prognostic biomarkers in HCC [5–7]. 

mir-33a is a highly conserved member of mir-33, which is located in the intronic region. It plays a role in lipid metabolism and provides glucose and cholesterol regulation. miR-33a has been shown to have growth, apoptosis, epithelial-mesenchymal transition, and tumor suppressor effects on cancer cells [8].

mir-203 serves as a tumor suppressor in many types of cancer including hepatocellular carcinoma, prostate, esophageal cancers, and breast cancer. miR-203 has been reported to play an important role in the carcinogenesis and progression of HCC, and there are also studies suggesting that miR-203 may be a prognostic factor in HCC [9,10].

The effects of miR‐361‐3p on proliferation, invasion, migration, and colony formation have been reported. A study has indicated that mir-361-3p, which has been shown to play an active role in body fluids, decreases the levels of expression in prostate secretion in prostate cancer patients[11].

mir-424, which has been found to have decreased expression in many cancer types, has been reported to play a role as tumor suppressor. It has been found to induce cell migration and play an important role in cancer development and progression in HCC [5].

In the literature, the expression levels of mir-33a, mir-203b, mir361-3p, and mir-424 have been reported to be either increased or decreased in various malignancies. A limited number of studies have reported these circulating miRNAs in cancer types. 

The current study examined for the first time the expression levels of mir-33a, mir-203b, mir361-3p, and mir-424 of peripheral blood serum in order to determine the potential biomarkers for early diagnosis, treatment, and prognosis in HCC.

## 2. Materials and methods

### 2.1. Sample collection

Venous blood samples were collected from 34 patients who were admitted to the Gastroenterology Outpatient Clinicin Haydarpaşa Numune Training and Research Hospital of the University of Health Sciences and İstanbul University Oncology Institute. They had been newly diagnosed with HCC and the patients who agreed to participate in the study were over the age of 18. The patients who were not included in the study were those who had received radiotherapy and chemotherapy,thecases with confirmed infection and chronic inflammatory diseases as well as those with tumors in another organ, and those who used antibiotics and antiinflammatory drugs and corticosteroids.

As the control group, blood samples were obtained from healthy individuals who were not related to any type of cancer. All venous blood samples were centrifuged at 3000 rpm for 10 min and the extracted serum samples were stored in a freezer at −80 °C. 

Drinking status, smoking status, and family history of cancer were recorded according to the declarations of patients and healthy individuals. The presence of chronic hepatitis B and C and cirrhosis was recorded according to laboratory and radiological test results. All epidemiologic variables and clinical data were collected by physicians (Table 1). 

**Table 1:** Epidemiologic variables and clinical data of HCC patients and healthy individuals.

Epidemiologic variables/Clinical data	Patients (n = 34, %)	Control (n = 34, %)	P value
Age (years) (mean ± SD)	51 ± 9	48 ± 10	P > 0.05
Female/male	10(29)/24 (71)	14 (41)/20 (59)	P > 0.05
Family history of cancer			
Yes	11 (32)	4 (12)	P < 0.05
Drinking status			
Never	27 (79)	30 (88)	P > 0.05
Ever	7 (21)	4 (12)	P > 0.05
Smoking status			
Never	26 (76)	23 (68)	P > 0.05
Ever	8 (24)	11 (32)	P > 0.05
Cirrhosis			
Yes	24 (71)	-	
Chronic hepatitis B	14 (41)	-	
Chronic hepatitis C	11 (32)	-	
Tumor size			
<5 cm	24 (71)	-	
≥5 cm	10(29)	-	
TNM stage			
I+II	22 (65)	-	
III+IV	12 (35)	-	
α-fetoprotein (ng/mL)			
<100	26 (76)	-	
≥100	8 (24)	-	

### 2.2. RNA isolation

RNAs were isolated from 500 µL fresh serum samples with a mirVana™ miRNA Isolation Kit (Thermo Fisher Scientific, Waltham, MA, USA; Catalog No AM1560) according to the manufacturer’s protocol. The concentration and purity of the isolated RNAs were measured at 230, 260, and 280 nm with a NanoDrop Spectrophotometer (ND-1000 Thermo Fisher Scientific). 

### 2.3. Reverse transcription and real time quantification of miRNA expression

From each sample of HCC patients and healthy individuals, 20 ng of total RNA was reverse‐transcribed to cDNA using specific miRNA TaqMan Assays and TaqMan MicroRNA Reverse Transcription Kit (Cat no: 4366596, Thermo Fisher Scientific) according to the manufacturer’s protocol[12]. TaqMan MicroRNA Assays hsa‐miR‐33a, hsa‐miR-203b, hsa‐miR-361-3p, hsa‐miR-424, and RNU6B (Cat No: PN4427975) were obtained from Applied Biosystems Thermo Fisher Scientific. RNU6B was used for endogenous control of miRNA expression analyses. 

The relative expression levels of miR‐33a, miR-203b, miR-361-3p, and miR-424 were evaluated with qRT‐PCR using TaqMan Universal PCR Master Mix (Cat no: 4364338, Thermo Fisher Scientific) with LightCycler 480 (Roche Applied Science, Penzberg, Germany). 

The RT‐qPCR was performed as follows: 1 cycle of 95 °C for 5 min, followed by 40 cycles of 95 °C for 10 s, 60 °C for 20 s, and 72 °C for 25 s. All the samples were run in duplicate. The relative quantification analysis was performed by the delta‐delta‐Ct method as described previously [13,14].

### 2.4. Statistical analysis

All the data were analyzed using SPSS Statistics version 15 for Windows. Two-sided Student’s t-test was used to determine the significance of the difference between the expression levels of the analyzed microRNAs. The threshold P value <0.05 was considered of statistical significance [13].Online tools were used for analyzing the power of the microRNAs in distinguishing HCC patients from healthy individuals and receiver operating characteristic (ROC) curves plotted [15].

## 3. Results

Table 1 presents the epidemiologic variables and clinical data of HCC patients and healthy individuals. 

The mean female patient age was 51 ± 9 and 29% and 41% of the healthy individuals were female with a mean age of 48 ± 10 years. More patients had a family history of cancer than healthy individuals (P < 0.05), which was determined as 32% and 12%, respectively.

In patients, 24% were ever smoker and 21% of them were ever drinker. In healthy individuals, 32% were ever smoker and 12% of them were ever drinker. As expected, most of the patients (71%) had cirrhosis and 41% had chronic hepatitis B, and 32% had chronic hepatitis C. None of the healthy individuals had chronic hepatitis B or C; also, none of them had cirrhosis. There was no significant difference between patients and healthy individuals in terms of sex, age, smoking, and drinking status (all P > 0.05).

**Figure 1:**
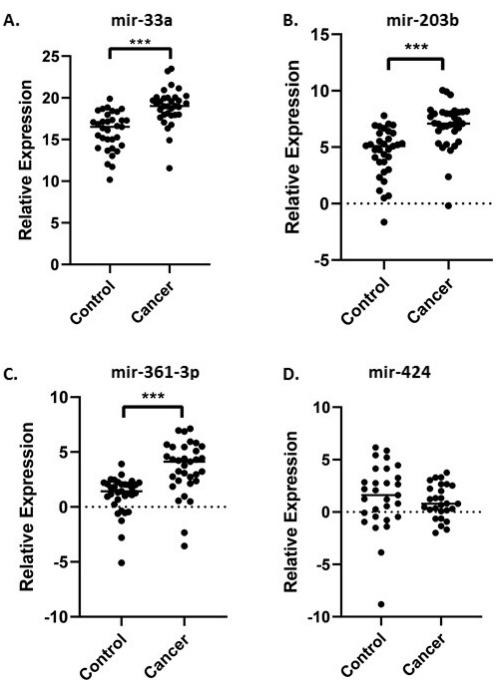
A. Expression of mir-33a between HCC patients (cancer) and the control group. B. Expression of mir-203 between the cancer and the control group. C. Expression of mir361-3p between the cancer and the control group. D. Expression of mir- 424 between the cancer and the control group. ***; P < 0.001.

The relative expression levels of cell-free miR‐33a, miR-203b, miR-361-3p, and miR-424 from serum samples were evaluated with RT‐qPCR. The Δ-ΔCt analysis of the RT‐qPCR data showed that mir-33a was 7.3-fold less expressed in HCC patients in comparison to healthy individuals (P = 0.000002), and mir-203b was less expressed like mir-33a.It was determined that mir-33a decreased 4.6-fold in patients when compared to the control group. Also, mir361-3p was 5.1-fold less expressed in patients than healthy individuals (P = 0.000032). These results indicated that the relative mir-33a, mir-203b, and mir361-3p expression levels were significantly decreased in HCC patients in comparison to the control group (P < 0.05) (Figures 1A–1C). When the RT‐qPCR data of mir-424 were evaluated, it was shown that there was not any significant change between the patient and control group (Figure 1D). 

When the RT‐qPCR results were compared, it was found that the miRNA of some HCC patients had more significant expression levels than other patients. It was found that the expression levels of mir361-3p and mir-424 were significantly decreased in female HCC patients in comparison to male HCC patients. Also, chronic hepatitis B and smoking were upregulated, and cirrhosis was downregulated compared to the other HCC patients (P < 0.05) (Table 2).

**Table 2:** Fold change in expression levels of miRNAs with epidemiological status of HCC patients.

	Patients/healthy individuals	HCC patients						
		Male/female	Hepatitis B yes/no	Hepatitis C yes/no	Smoking yes/no	Drinking yes/no	Cirrhosis yes/no	Family historyof cancer yes/no
Mir-33a	7.3↓*	↔	2 ↓*	1.3 ↓*	2.8 ↓*	↔	↔	↔
Mir-203b	4.6 ↓*	↔	2 ↓*	↔	2.6 ↓*	↔	1.2 ↑*	↔
Mir361-3p	5.1 ↓*	2.2 ↓*	1.6 ↓*	↔	3.7 ↓*	↔	1.7 ↑*	↔
Mir-424	↔	1.9 ↓*	1.1 ↓*	↔	2.4 ↓*	↔	↔	↔

*; P < 0.05; ↑, upregulate; ↓, downregulate; ↔, no difference.

**Figure 2:**
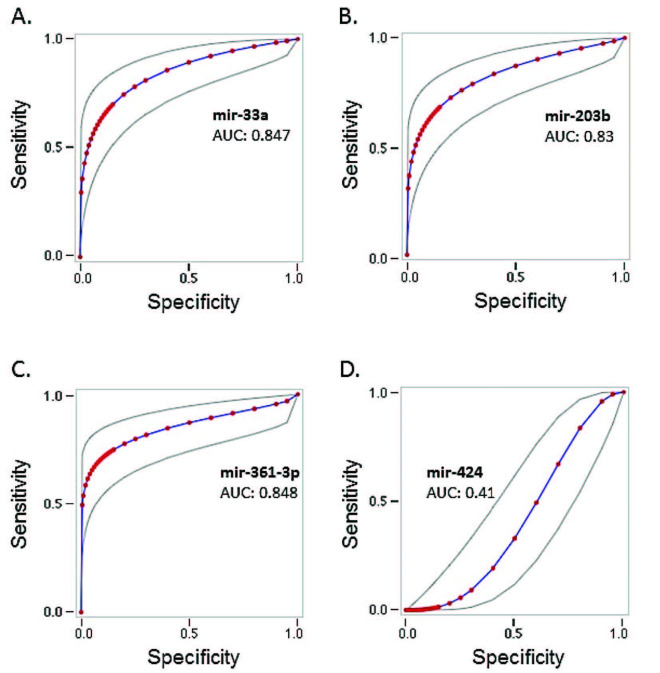
A. Receiver operating characteristic (ROC) analysis curve of mir-33a between HCC patients (cancer) and the control group. B. ROC curve of mir-203 between the cancer and the control group. C. ROC curve of mir361-3p between the cancer and the control group. D. ROC curve of mir-424 between the cancer and the control group. Curves to see the power of miRNAs in distinguishing HCC patients from healthy individuals.

We plotted ROC curves for analyzing the power of the microRNAs in distinguishing HCC patients from healthy individuals. It was found that mir361-3p had the highest area under curve (AUC; 0.848), while mir-33a, mir-203b, and mir-424 had AUC values of 0.847, 0.83, and 0.43, respectively, in comparison to healthy individuals (Figures 2A–2D).

## 4. Discussion 

In this study, we demonstrated that mir-33a, mir-203b, mir361-3p, and mir-424 had variable but significantly reduced expression levels compared to HCC tumor samples and healthy control serum. It is thought that the causes of different miRNA expression profiles in cancer patients may be passive or active release of miRNAs by tumor cells. The expression levels of these 4 miRNAs in the serum of HCC patients were observed to be significantly reduced. It is known that miRNAs in body fluids can survive without degradation for a long time and are not affected by changing pH and temperature levels [16].

miR-33a plays a crucial role in regulation of cholesterol metabolism and cellular phenotypes associated with carcinoma progress. miR-33a suppresses cell proliferation and metastasis of some cancer types by targeting oncogenes [17,18]. Downregulation of miR-33a activity in HCC increases the proliferative and invasive potential of HCC cells. The data obtained from the study also show that miR-33a plays a tumor suppressor role in HCC. The mean 7.3-fold decrease in miR-33a was observed in HCC patients.

Studies show that miRNA-203 acts as a tumor suppressor in various types of cancer. It was shown that mir-203 has a critical role in cell proliferation and invasion in prostate carcinoma [19]. Also, it was demonstrated that mir-203 expression levels were significantly lower in colorectal cancer tissues in comparison to nontumor tissues and it has overexpression in ovarian cancer [9,20]. These results show that the expression levels of mir-203 differ in each carcinoma. Our results show that mir-203 expression levels decreased 4.6-fold in patients in comparison to the control group. The results show similarity with colorectal carcinoma.

The effects of mir361‐3p on proliferation, invasion, migration, and colony formation have been reported. mir361-3p, which plays an active role in body fluids, has been shown to decrease the levels of expression in prostate secretion in prostate cancer patients. Chen et al. reported that mir361-3p is upregulated in NSCLC and it was shown that it inhibits cell proliferation, migration, and invasion [11]. Another study indicated that the overexpression of mir361-5p suppresseslung cancer proliferation and invasion and it acts as a tumor suppressor in lung cancer [21]. Our results show that mir361‐3p was downregulated and decreased 5.1-fold in patients in comparison to the control group.

A decreased expression of mir-424 has been shown in HCC cell lines and primary tumors, while decreased mir-424 has been reported to accelerate cell proliferation, migration, and invasion. In addition, the significant role of c-Myb, an important invasive molecule in HCC, is a direct target of mir-424 in tumorigenesis [5]. In our study, there is no significant expression level of mir-424 in serum samples obtained from HCC patients in comparison to healthy individuals.

There are significant sex differences in the risks and outcomes of cancer between females and males [22]. Our study showed that there are differences in microRNA (miRNA) expression in adult male and female HCC patients. The expression levels of mir361-3p and mir-424 were significantly decreased in female patients in comparison to male patients. Also, drinking and smoking status, chronic hepatitis B, and cirrhosis were significantly associated with circulating miRNA expression [6]. 

According to our results, there is no correlation with the expression levels of mir-203b, mir361-3p, and mir-424 in patients withchronic hepatitis C. However,the expression levels of mir-33a, mir-203b, mir361-3p, and mir-424 were decreased in patients with chronic hepatitis B. It was shown that mir-33a, mir-203b, mir361-3p, and mir-424 were upregulated in smokers, and mir-203b and mir361-3p were downregulated in cirrhosis patients compared to other HCC patients. Despite these results, when all data of HCC patients were compared with healthy individuals, it was obvious that mir-33a, mir-203b, and mir361-3p were less expressed (P < 0.05).

The ROC curve results of mir361-3p, mir-33a, and mir-203b show that they are sufficient to have the power to differentiate HCC patients from healthy individuals. Since the AUC value of mir-414 was 0.43, it was determined that there was no significant difference between the groups.

## 5. Conclusion

In conclusion, this was the first study that examined the expression levels of mir-33a, mir-203b, mir361-3p, and mir-424 of peripheral blood serum in order to determine potential biomarkers for early diagnosis, treatment, and prognosis in HCC. It was determined that mir-33a (fold change: 7.3 and P < 0.001), mir-203b (fold change: 4.6 and P < 0.001), and mir361-3p (fold change: 5.1 and P < 0.001) was less expressed than healthy individuals and mir-424 did not show any significant change between the patient and control group. mir-33a, mir-203b, and mir361-3pmaypotentially be used as noninvasive biomarkers for HCC diagnosis and treatment in the future.

## Ethics committee approval

The study was approved by the Ethics Committee of İstanbul University (document number 2016/1297).
